# Multi-Antigen Imaging Reveals Inflammatory DC, ADAM17 and Neprilysin as Effectors in Keloid Formation

**DOI:** 10.3390/ijms22179417

**Published:** 2021-08-30

**Authors:** Mathias Rath, Alain Pitiot, Michael Kirr, Waltraud Fröhlich, Bianca Plosnita, Stefan Schliep, Jürgen Bauerschmitz, Andreas S. Baur, Christian Ostalecki

**Affiliations:** 1 Department of Urology, University Hospital Heidelberg, 69120 Heidelberg, Germany; mathias.rath@med.uni-heidelberg.de; 2 Laboratory of Image & Data Analysis, Ilixa Ltd., London W1U 6NQ, UK; alain.pitiot@tissuegnostics.com; 3 Department of Dermatology, Universitätsklinikum Erlangen, Friedrich-Alexander-University Erlangen-Nürnberg, 91054 Erlangen, Germany; kirr@students.uni-marburg.de (M.K.); waltraud.froehlich@uk-erlangen.de (W.F.); stefan.schliep@uk-erlangen.de (S.S.); juergen.bauerschmitz@uk-erlangen.de (J.B.); andreas.baur@uk-erlangen.de (A.S.B.); 4 Deutsches Zentrum Immuntherapie (DZI), 91054 Erlangen, Germany; 5 Comprehensive Cancer Center (CCC) Erlangen, 91054 Erlangen, Germany; 6 TissueGnostics GmbH, 1020 Vienna, Austria; bianca.plosnita@tissuegnostics.com; 7 Department of Psychiatry and Psychotherapy, Universitätsklinikum Erlangen, Friedrich-Alexander-University Erlangen-Nürnberg, 91054 Erlangen, Germany

**Keywords:** scar, dendritic cells, tumor microenvironment, single-cell analysis, multiplex imaging, proteases, tumor biology

## Abstract

Keloid is an aberrant scarring process of the skin, characterized by excessive extracellular matrix synthesis and deposition. The pathogenesis of this prevalent cutaneous disorder is not fully understood; however, a persistent inflammatory process is observed. To obtain more insight into this process, we analyzed lesional, perilesional and healthy tissue using multi-antigen-analysis (MAA) in conjunction with a data mining approach. Here, we demonstrate that monocyte-derived inflammatory dendritic cells (CD1a+, CD11c+, CD14+) and activated CD4+ T lymphocytes (CD45 RO+) dominated the immune infiltration in keloids while associating with fibroblasts. In perilesional tissue, precursor immune cells were dominant in the perivascular area, suggesting that they were attracted by an immune process, potentially in the lesional area. Supporting this hypothesis, only in keloid lesions, high levels of ADAM10/17 and Neprilysin (CD10) were observed in both fibroblasts and leukocytes. The spatial proximity of these two cell types, which could be confirmed by image analysis only in lesional tissue, could be a potential factor leading to the activation of fibroblasts. Our findings provide new insight into the pathogenesis of keloid formation and reveal metalloproteinases as a target for therapeutical intervention.

## 1. Introduction

The formation of scar tissue after injury constitutes a physiological healing process, reestablishing the functionality of the skin. Depending on the type of tissue and the type of injury, the wound healing process may vary. Tissue may regenerate with or without scarring, or tissue fibrosis may lead to a pathological scar [1,2]. Under some circumstances, the healing process becomes excessive, resulting in abnormal scar formation termed keloids (KD) [3]. Histopathological KD is characterized by a thick and flattened epidermis, increased cellularity and an abundance of extracellular matrix deposition in the dermis [4], resulting in pain, pruritus and aesthetic impairment [5]. Likewise, important biological functions, such as normal tissue growth, thermoregulation or normal range of movement, are impaired [6].

Fibroblasts are believed to have the most critical role in keloid formation [7], as they show an increased proliferation rate and ECM production, leading to a high cellular density and typical dense tissue texture [7,8,9]. Although their pathological role is unquestioned, the mechanism by which fibroblasts are seemingly continuously stimulated remains unclear [10]. Mast cells are considered to play a role in every phase of wound healing. This includes the proliferative phase, in which angiogenesis, as well as keratinocyte and fibroblast activity, is stimulated, and the scar formation and remodeling phase, in which fibroblasts are involved [11].

The presence of antigen-presenting cells such as dendritic cells (DC), Langerhans cells and macrophages in keloid tissue [12,13], have suggested an ongoing immune reaction and/or inflammatory reaction as the underlying cause of KD formation [14]. Active immune cells release an array of cytokines and growth factors, potentially leading to increased fibroblasts proliferation and excessive extracellular matrix (ECM) deposition [7,15]. Supporting this conclusion, Langerhans cells, DCs, lymphocytes and macrophages are among the most common inflammatory cells found in keloid tissue [7,15,16,17]. The high concentration of lymphocytes correlates with an increased recurrence rate due to a strong inflammatory response [18]. These findings could point to an antigen-driven process, an assumption that is supported by the presence of high numbers of CD45+ T memory cells [19]. 

Most immunological effectors, including cytokines and chemokines, are expressed as inactive precursors and need to be processed by an activated protease. In this context, ADAM10 and 17 are of particular importance for cellular differentiation and proliferation, cleaving such important mediators such as proTNF and the Notch receptors [20]. Hence, they are involved in many pathophysiological conditions, including atopic eczema and psoriasis [21,22]. These proteases cleave their substrates at the plasma membrane [23] but also in endosomal compartments [24]. In addition, they are packaged into extracellular vesicles for transfer into target cells by secretion [25] or by cell-to-cell contact [26]. In addition to ADAMs, other metalloproteases such as Meprin are thought to play an important role in the processing of procollagens and the subsequent formation of collagen fibrils leading to fibrosis [27,28]. Hence, these proteases have a wide range of activity locally and in the cellular environment.

To understand these pathophysiological processes, the identification, characterization and quantification of immune cells and associated factors are of paramount importance. Typically, for immune histological tissue analyses, the tissue is stained with one antibody without quantifying the antigen. We have employed a multi-antigen analysis approach described previously [29] in conjunction with imaging software, permitting the spatial assessment and quantification of more than 100 antigens on one single tissue section. This allows more detailed insight into pathological processes in tissue, as demonstrated previously [26,30]. Here, we used this technology to examine the immune infiltration and marker expression in keloid tissue. While monocyte-derived inflammatory dendritic cells (DC) were the main infiltrating immune cell population, the striking upregulation of ADAM10, ADAM17 and Neprilysine in lesional fibroblasts may point to a prominent role of these proteases in the pathogenesis of keloids. Hence, these proteases may represent a promising therapeutic target.

## 2. Results

### 2.1. Lesional Immune Cells Associate with Fibroblasts

For our study, we analyzed eight lesional and corresponding perilesional regions from primary keloid formations of the upper chest. For comparison, five samples from healthy individuals were obtained and analyzed from the same anatomical region (Figure S1a). In order to assemble a set of antibodies for the MAA with a strong and specific staining pattern, we screened over 500 antibodies on keloid tissue. This unbiased approach yielded 58 antibodies, including 45 immune cell markers, 5 structural markers and 8 markers for proteases and their substrates (Figure S1b).

**Figure 1:**
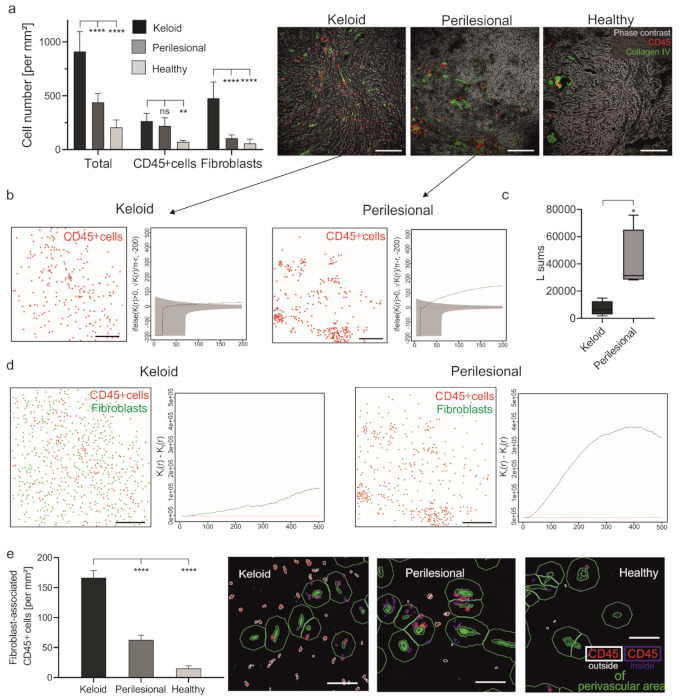
The increased number of fibroblast-associated leukocytes in keloid. (**a**) Skin sections of keloid, perilesional and control tissue were subjected to an MAA analysis, and the number of total cells and CD45+ cells were quantified (left panel). Representative images showing an overlay of phase contrast (grey) and CD45+ cells (red) in addition to blood vessels (collagen type IV, green) (right panel). Scale bars = 200 μm; Data are means ± SEM from n patients (*n* (Keloid) = 8, *n* (perilesional) = 8, *n* (control) = 5). Statistical significance was assessed based on the *p* value (ns *p* > 0.5, ** *p* < 0.01, **** *p* < 0.0001) and determined using, two-way ANOVA and Dunnett’s test. (**b**) Characterization of the spatial distribution of CD45+ cells (red) across analyzed keloid (*n* = 4) and perilesional (*n* = 4) tissue samples. Besag’s L function (solid black line) and acceptance envelope (grey), which give the range of L values for which the cell distribution is not significantly different from complete spatial randomness (red dotted line). For the keloid sample, Besag’s L function is either engulfed in the envelope or hovering just above it, indicating that the cells are mostly randomly dispersed. For the perilesional sample, the Besag’s L function is markedly above the envelope, indicating that the cells are very much clustered. Scale bars = 200 μm. (**c**) Sum of the Besag’s L functions. *n* (Keloid) = 4, *n* (perilesional) = 4, Data are means ± SEM, * *p* < 0.05. (**d**) The evaluation of the spatial association of immune cells and fibroblasts. Cell plots showing the distribution of CD45+ cells (red dots) and fibroblasts (green dots) in keloid (*n* = 4) and perilesional (*n* = 4) tissue areas and a combination of two Ripley’s K functions (Kii—Kij, for i = CD45 and j = fibroblast) that characterize the spatial segregation of these two cell types. Scale bars = 200 μm. (**e**) By using the StrataQuest software, a perivascular area was determined by using the collagen type IV staining (green cells) surrounded by a ring (green) with an exterior radius of 20 μm. The number of CD45+ cells outside the perivascular area (defined in the following with the term ‘fibroblast-associated cells’; red cells with ring) were measured. Scale bars = 40 μm; Data are means ± SEM from *n* patients (*n* (Keloid) = 8, *n* (perilesional) = 8, *n* (control) = 5). **** *p* < 0.0001, one-way ANOVA and Tukey’s test.

Since keloid formation is associated with cellular infiltration [10], we first wanted to assess the numbers of two cell populations (cell/mm^2^ tissue) involved in keloid formation, namely immune cells (CD45+) and fibroblasts (Fibroblast+ (recombinant antibody from Miltenyi Biotec), Vimentin+, CD45-). The overall cell density in keloids (≈900 cells/mm^2^) was 2.1-fold higher compared to the perilesional area (≈ 440 cells/mm^2^) and 4.3-fold higher compared to healthy tissue (≈210 cells/mm^2^) (Figure 1a).

The density of immune cells in keloids was only about 20% higher in lesional (≈270 cells/mm^2^) than perilesional (≈220 cells/mm^2^) regions, but both regions exhibited a significantly higher density than healthy tissue (≈70 cells/mm^2^) (Figure 1a). However, since the number of fibroblasts was 4.4-fold lower lesionally (≈110 cells/mm^2^) than perilesionally (≈480 cells/mm^2^), also the ratio of immune cells to fibroblasts was lower in lesional regions (1:1.8) as compared to perilesional areas (1:0.47) and healthy tissue (1:0.83).

This result was surprising at first but could be explained by the differences in the spatial distribution of immune cells (CD45+). As illustrated in Figure 1a (images), Figure 1b and Figure S2 (cell plots and Besag’s L function), immune cells in keloid lesions were seemingly randomly distributed as compared to perilesional areas, where they clustered together. This observation was confirmed by comparing the sum of the values of Besag’s L function (Figure 1c), which describes the spatial distribution of a given cell population, indicating that the cells are clustered or randomly dispersed.

The perilesional and lesional areas also differed with respect to the relative spatial distribution of immune cells and fibroblasts. In perilesional areas, immune cells showed a tendency to cluster and were found in larger distance to fibroblasts, while lesional immune cells are in closer proximity to fibroblasts (Figure 1d and Figure S3, cell plots). This observation was confirmed using the Ripley K function that quantified the spatial segregation of immune cells and fibroblasts (Figure 1d and Figure S3, graphs). Additionally, immune cells located outside the perivascular area, i.e., spatially more closely associated with fibroblasts than vascular immune cells, were quantified. This was determined by counting immune cells inside and outside of a 20 μm spatial ring around these blood vessels (characterized by: collagen type IV+ cells). Lesional immune cells were significantly more located outside the perivascular area as compared to immune cells in perilesional or healthy tissue (≈167 vs. ≈63 vs. ≈15 cells/mm^2^) (Figure 1e).

Cells detected outside the perivascular area will be referred to by the term fibroblast-associated immune cells in the further course. These data confirmed previous findings suggesting an interaction of immune cells and fibroblasts, potentially leading to their activation [31]. Furthermore, the perivascular clustering of immune cells in perilesional areas could point to the extravasation of these cells, potentially attracted by lesional immune events.

### 2.2. DCs Are the Predominant Cell Type in Keloid Tissue

In order to determine and quantify the immune cell subtypes present in keloid tissue, we analyzed the following marker combinations: CD4+ T cells (CD3+, CD4+), CD8+ T cells (CD3+, CD8+), B cells (CD20+), NK cells (CD3−, CD56+), dermal DCs (CD1a+, CD11c+), monocytes/macrophages (Mo/MΦ) (CD14+, CD68+) and mast cells (CD117+). A given marker combination was identified by co-localization analysis, using imaging software (StrataQuest, TissueGnostics, Vienna, Austria).

**Figure 2:**
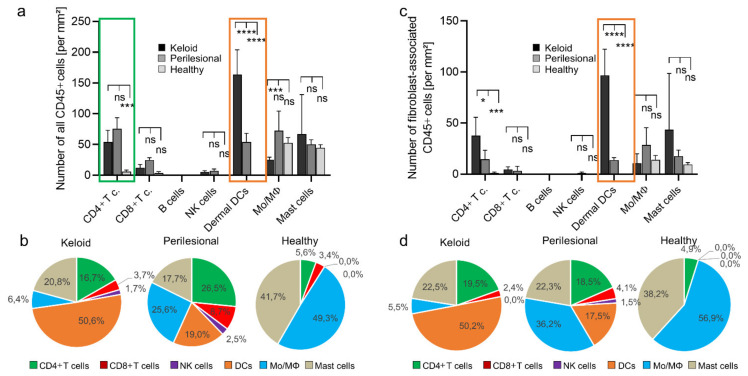
A high abundance of dermal dendritic cells in keloid. (**a**) The entire population of CD45+ cells was subclassified and subsequently quantified for keloid, perilesional and control tissue. Immune subsets were characterized by following marker combination: CD4+ T cells (CD3+, CD4+), CD8+ T cells (CD3+, CD8+), B cells (CD20+), NK cells (CD3-, CD56+), dermal DCs (CD1a+, CD11c+), monocytes/macrophages (Mo/MΦ) (CD14+, CD68+), mast cells (CD117+). (**b**) Pie charts showing the relative abundance distribution of immune subsets in keloid, perilesional and control tissue, classified in (**a**). Each pie slice represents a distinct immune subtype. (**c**) The fibroblast-associated CD45+ cell population determined in Figure 1 was divided in different leukocyte subtypes (see Figure 2a) and quantified for keloid, perilesional and control tissue. (**d**) Pie charts of the relative distribution of immune cells classified in (**c**). Each pie slice represents a certain immune subtype. Data are means ± SEM from *n* patients (*n* (Keloid) = 8, *n* (perilesional) = 8, *n* (control) = 5). Statistical significance was assessed based on the *p* value (ns *p* > 0.5,* *p* < 0.05, *** *p* < 0.001, **** *p* < 0.0001) and determined using two-way ANOVA and Tukey’s test.

A quantification revealed different distribution patterns of immune cell populations in lesional, perilesional and healthy tissue (Figure 2a (cell numbers) and Figure 2b (relative abundance of cells in %)). Notably, a high number of dermal DCs (≈160 cells/mm^2^) was measured in keloid tissue, representing more than 50% of all CD45+ cells, whereas far fewer dermal DCs were measured in the perilesional areas (≈55 cells/mm^2^; 19.0% of leucocytes, Figure 2a, orange box). Conversely, no dermal DCs were found in healthy tissue. There, the most prominent cell populations were monocytes/macrophages (≈55 cells/mm^2^; 49.3% of immune cells), whereas in keloid tissue, monocyte/macrophage density was only at ≈25 cells/mm^2^ (6.4% of immune cells). Perilesional tissue revealed an intermediate phenotype with elevated numbers of dermal DCs (≈55 cells/mm^2^; 19.0%) and a reduced monocyte population (≈70 cells/mm^2^; 25.6%) compared to healthy tissue. No significant differences could be observed in the number of mast cells between lesional, perilesional and healthy tissue (≈67 vs. ≈49 vs. ≈45 cells/mm^2^).

In line with the increased presence of DC in keloid tissue, we recorded higher levels of CD4+ lymphocytes (≈55 cells/mm^2^; 16.7%). However, even higher numbers were found in perilesional tissue (≈75 cells/mm^2^; 25.6%) but not in healthy tissue (Figure 2a, green box). Other lymphocyte populations (CD8+ cell, B cells and NK cells) had seemingly a minor role in absolute numbers and relative abundance. In summary, dermal DCs and CD4+ T cells were the most abundant cell population in keloid tissue.

Next, we asked whether these increased numbers of immune cells were associated with fibroblasts. Indeed, DCs and also CD4+ cells associated predominantly with fibroblasts as assessed and quantified as described above (Figure 1e). In particular, DCs were significantly more associated with fibroblasts in lesional areas as compared to perilesional areas or healthy tissue (≈100 vs. ≈ 5 vs. ≈0 cells/mm^2^) (Figure 2c, orange box), comprising 50.2% vs. 17.5% vs. 0% of all dermal DC cells (Figure 2d, orange). Conversely, little difference was seen with monocytes associated with fibroblasts in low numbers (Figure 2c). Interestingly, their relative association with fibroblasts decreased significantly from healthy to lesional areas (56.9% vs. 36.2% vs. 5.5%) (Figure 2d, blue).

Fibroblast-associated CD4+ cells were likewise more abundant in lesional as compared to perilesional tissue (≈40 vs. ≈15 cells/mm^2^), even though the amount of all CD4+ T cells in the analyzed tissue was higher in perilesional areas (≈75 vs. ≈55 cells/mm^2^) (Figure 2c). Again, this may have been due to perivascular clustering as part of an extravasation effect. The average number of mast cells was about two–four-fold higher in lesional tissue compared to perilesional and healthy tissue (≈43 vs. ≈18 vs. ≈9 cells/mm^2^). However, due to great differences inter-individually and analyzed tissue areas, these differences were not significant.

In summary, DCs and CD4+ T cells were associated with fibroblasts at a significantly high proportion in keloid tissue when compared to perilesional or healthy tissue.

### 2.3. Lesional DCs Have an Inflammatory Phenotype

**Figure 3:**
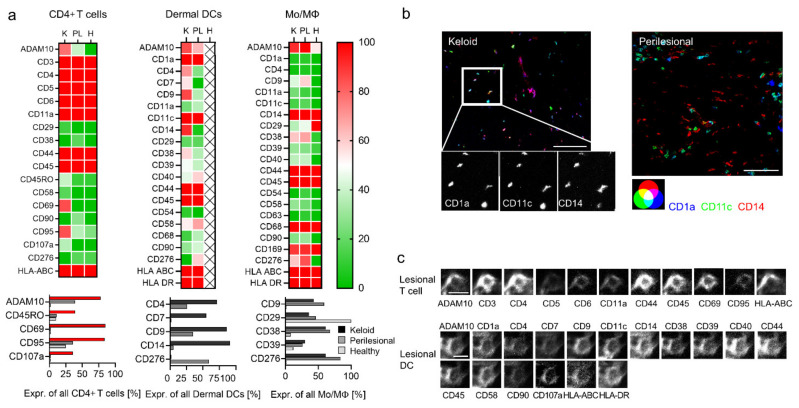
Deep phenotyping reveals activated T cells and inflammatory DCs in keloids. (**a**) Deep phenotyping of immune subtypes in the keloid (K), perilesional (PL) and control (H) skin sections are shown for indicated markers in heat maps (upper panel). The color code from red (100) to green (0) displays the expression intensity of specific antigens in percentage in all cells of a certain immune subtype. Markers showing major differences in their expression intensity are depicted separately in a bar diagram (lower panel). (**b**) Fluorescence co-localization images for CD1a (blue), CD11c (green), CD14 (red), the characteristic markers for inflammatory DCs. Close-up images of single markers indicate the triple expression in each cell in the keloid sample. Scale bars = 50 μm. (**c**) Representative protein profile of individual cells (lesional T cell and lesional DC) in keloid tissue by MAA. The two panels show the same section, stained with 11 and 17 antibodies, respectively. Scale bars = 10 μm.

In order to support our assumption of an active immune reaction in keloid tissue, we analyzed additional immune markers reflecting the identity and activation status of the cells. The results are displayed in heat maps showing the average expression level for each maker analyzed in CD4+ T cells, DCs, monocytes/macrophages (Figure 3a, heat maps), CD8+ T cells and NK cells (Figure S4). The most noticeable differences were quantified and displayed in bar diagrams for each cell population (Figure 3a, graphs).

Even though there was no significant quantitative difference of CD4+ T lymphocytes in lesional and perilesional tissue (see Figure 2a, green box), some differentiation and activation markers revealed increased expression in keloid lesions, namely ADAM10, CD45RO, CD69, CD95 and CD107a (Figure 3a, red bars). Together, this suggested the presence of activated memory T cells. Conversely, other lymphocyte subsets (CD8+ cell and NK cells) displayed a comparable marker phenotype in keloid as well as perilesional tissue (Figure S4).

Almost all DCs in lesional areas co-expressed CD14 besides CD1a and CD11c (CD1a+, CD11c+, CD14+, Figure 3a). Hence, they could potentially be considered as DC3 [32] or inflammatory monocyte-derived DCs [30]. MMA-independent conventional immunohistochemistry staining confirmed the increased appearance of these makers in lesional tissue (Figure S5). Conversely, in perilesional tissue, these markers differentiated two different cell populations, namely CD1a+ CD11c+ CD14− cells (Figure 3b, cyan-colored), likely dermal DCs, and CD1a− CD11c− CD14+, likely monocytes/macrophages (red-colored). Additional surface markers, including CD4, CD7 and CD9, were also found on lesional DCs (Figure 3a), potentially a sign of further differentiation. Figure 3c shows two representative MAA protein profiles of a lesional T cell and DC.

The expression profile of monocytes/macrophages was similar in keloid and in perilesional tissue and showed only differences in comparison with monocytes/macrophages in healthy skin. The increased levels of markers such as CD9, CD38 and CD39 in keloid and perilesional tissue potentially indicated that also these cells had started to differentiate (Figure 3a).

Taken together, the most prominent cell population in lesional areas potentially represented a DC3 cell or monocyte-derived inflammatory DC.

### 2.4. Elevated Levels of Metalloproteases and Their Substrates in KD

So far, our data implied an activated immune reaction in keloid tissue that might influence the activation and proliferation rate of fibroblasts. To substantiate this assumption, we were looking for additional evidence. Since the identification of smaller soluble factors, such as cytokines, is not possible by MAA, we focused on the presence of representative metalloproteases, which are upstream activators of proinflammatory and differentiation molecules as well as signaling pathways [20]. Furthermore, ADAM10 is the major ephrin-B2 sheddase in fibroblasts leading to their activation [33].

**Figure 4:**
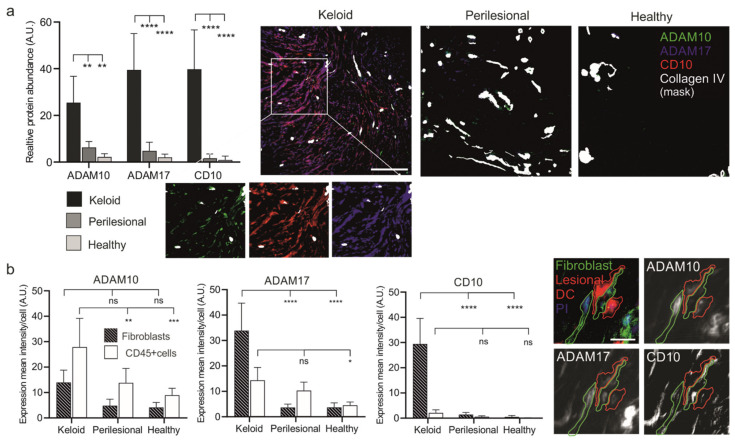
Increased expression levels of metalloproteases in keloid patients. (**a**) Tissue sections of keloid, perilesional and control skin were systematically analyzed for metalloproteinase expression by MAA. Quantification of the different metalloproteases (left panel). Relative expression levels of proteases were determined by assessing the grey value intensity. The respective stainings are shown in co-localization images representing an overlay of four markers (ADAM 10, ADAM17, CD10 and a Collagen type IV-mask). Individual close-up images for KD visualize the expression of the proteases. Scale bars = 200 μm. (**b**) Mean expression levels of ADAM 10, ADAM17 and CD10 in fibroblasts and CD45+ cells. MAA-image gallery showing the expression of analyzed proteases in fibroblasts (green encircled cells) and inflammatory DCs (red encircled cells) in keloid tissue. Fibroblasts were defined by using anti-fibroblast antibody plus CD45 negative signal, and lesional DCs by anti-CD1a, anti-CD11c and anti-CD14 antibodies. Scale bars = 10 μm. Data are means ± SEM from *n* patients (*n* (Keloid) = 8, *n* (perilesional) = 6, *n* (control) = 5). Statistical significance was assessed based on the *p* value (ns *p* > 0.5, * *p* < 0.05, ** *p* < 0.01, *** *p* < 0.001, **** *p* < 0.0001) and determined using two-way ANOVA and Tukey’s test.

The three proteases we analyzed, ADAM10, ADAM17 and CD10 (Neprilsyin), were strongly upregulated in lesional as compared to perilesional or control tissue (Figure 4a, Figure S6). In particular, CD10 was found almost exclusively in keloid lesions. The expression of these proteases in endothelial cells was excluded (collagen IV mask, white), as ADAM10 is intrinsically upregulated in these cells.

Next, the presence of these proteases was assessed in fibroblasts and immune cells (Figure 4b). Immune cells (CD45+) revealed a stepwise increase of ADAM10 and 17 expressions from healthy to lesional areas, while CD10 was almost undetectable in any tissue area. Conversely, fibroblasts showed a strong increase of ADAM17 and an almost dramatic appearance of CD10 (Figure 4b) but only in lesional areas.

**Figure 5:**
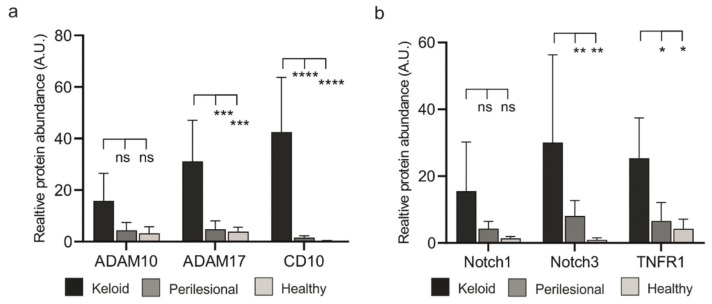
Lesional fibroblasts show elevated expression of proteases and their substrates (**a**) Evaluation of expression of ADAM10, ADAM17 and CD10 in fibroblasts across analyzed keloid and perilesional tissue samples. (**b**) Quantitative analysis of the different substrate of metalloproteases by MAA. Relative expression levels of Notch1, Notch3 and TNFR1 in keloid, perilesional and control tissue. Data are means ± SEM from *n* patients (*n* (Keloid) = 8, *n* (perilesional) = 6, *n* (control) = 5). Statistical significance was assessed based on the *p* value (ns *p* > 0.5, * *p* < 0.05, ** *p* < 0.01, *** *p* < 0.001, **** *p* < 0.0001) and determined using two-way ANOVA and Tukey’s test.

The elevated protease levels in keloid fibroblasts coincided with close proximity to CD45+ immune cells, as ADAM10, ADAM17 and CD10 reveal a high expression predominantly found in lesional fibroblasts (Figure 5a).

It is not clear how the close proximity of CD45+ cells and fibroblasts could lead to a higher number of fibroblasts. We speculated that activated proteases would process ligand precursors that could stimulate cellular receptors, including TNFR1 (ADAM17 substrate) and the Notch receptor family (ADAM10 substrate). To test this assumption, we assessed protein levels of Notch receptors and TNFR1. MAA analyses revealed an increased abundance of Notch proteins and TNFR1 in keloid tissue (Figure 5b and Figure S7a). Notably, these proteins co-localized with the respective proteases (Figure S7b).

Taken together, these data could hint at a functional interaction of activated immune cells with fibroblasts in keloid lesions.

## 3. Discussion

We have previously demonstrated that multi-antigen analysis (MAA) of tissue sections may provide new insights into the pathogenesis of diseases. This conclusion is supported by the here-presented study, revealing specific cell types, factors and potential events leading to the development of keloids. Our results suggest that the immune infiltration in keloids is dominated by inflammatory dendritic cells and memory T cells. These cells are associated closely with fibroblasts expressing high levels of ADAM17 and CD10. The expression of these proteases coincided with the appearance of Notch receptors in fibroblasts, which are substrates of ADAM10. ADAM10 was mainly found in activated immune cells. Taken together, activated immune cells may stimulate fibroblasts for increased activity and proliferation rate.

Our findings are in general agreement with previous work on the pathogenesis of keloids, proposing an inflammatory process in the dermis involving fibroblasts, immune cells and eventually keratinocytes [4]. Additionally, the presence of proteases, namely MMP 1, 2, 3, and 9, had been described [4]. However, the exact etiology of keloid scarring remained unknown. While recent studies have identified many inflammatory factors, among them TGF-beta, IL-6 and TNF alpha, a detailed sequence of events has not been described yet [34].

Particularly notable was the appearance of proteases in fibroblasts and leukocytes only in lesional areas. This would agree with an ongoing immune activation event, as these proteases induce cellular differentiation and signaling events by cleaving different precursor molecules. Particularly ADAM17 has been associated with inflammation, as the protease activates TNF alpha. Conversely, ADAM10 seems more involved in cellular differentiation and proliferation [35]. Recently, ADAM10 was shown to induce ephrin-B2 shedding, promoting fibroblast activation [33]. Taken together, it is conceivable to assume that these proteases are involved in the wound healing process [36] and the activation of fibroblasts [37]. For reasons that are not clear, this process seems to continue excessively, leading to keloid formation. To what extent the protease activity in keloid tissue differs from physiological wound healing and to what extent the proteases play a major role in keloid development at all still needs to be investigated. We would hypothesize that inflammatory DCs are an important driver of this process, potentially acting in trans on the cellular environment. We have previously demonstrated such a mechanism, demonstrating the activation of keratinocytes by melanoma cells in the early tumor microenvironment [26]. This occurred through a transfer of ADAM10-activating SPPL3 through melanoma cell dendrites connecting with keratinocytes. Our technology did not have the necessary resolution power to detect such putative interactions in primary tissue. In addition, there is increasing evidence that extracellular vesicles secreted by activated cells such as DCs, transfer effector proteins to the microenvironment [38].

This leads to the question of why inflammatory DCs appear or develop in keloids. Usually, the appearance of such cells correlates with an immune reaction driven by an antigen [39,40]. The appearance or development of activated memory T cells in keloid lesions, as shown here, would support such an assumption and could also explain why corticosteroid injections have a prominent role in keloid treatment. A putative antigen could be incorporated by DCs in a soluble form and subsequently cross-presented. For example, the excessive activity of several proteases could produce a novel peptide from an unknown precursor protein, essentially generating an autoimmune antigen. However, this remains speculation.

**Figure 6:**
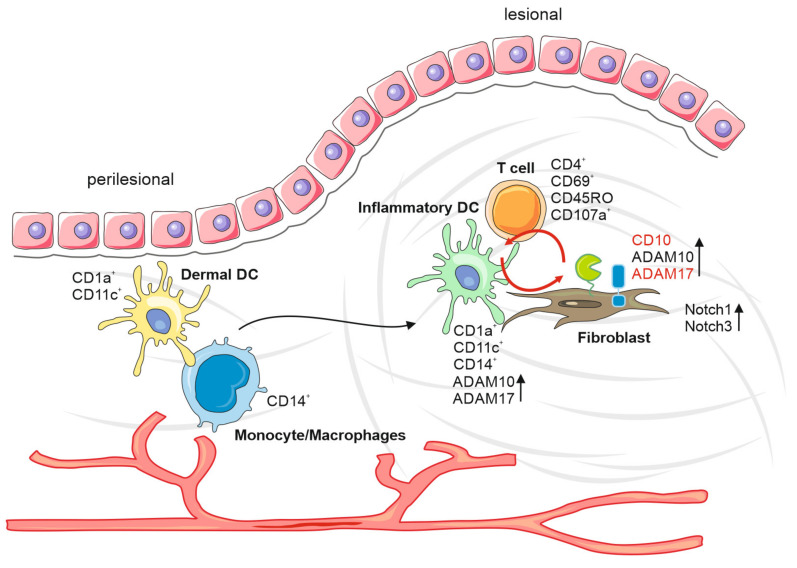
Keloid formation model. Increased DC precursor cells from perilesional vessels, attract by autoimmune antigens during the wound healing process, migrate to lesional areas and develop into inflammatory DCs. The interaction between inflammatory DCs, T cells, monocytes and fibroblasts results in an increased protease expression. This inflammatory environment leads to increased expression of proteases and their precursor effectors, such as the Notch receptors, leading to several stimulating effects, including the proliferation of fibroblasts and production of extracellular matrix components.

In summary, we could envision the following different scenario(s) (Figure 6). Potentially attracted by the appearance/generation of an autoimmune antigen during the wound healing process, DC precursor cells, likely monocytes, extravasate from perilesional vessels, migrate to lesional areas and develop into inflammatory DCs. The infiltration and differentiation of T cells, mostly of the memory phenotype, supports this scenario, as well as the decrease of relative numbers of monocytes. Alternatively, activated inflammatory DCs may attract memory T cells without being stimulated by an antigen. For reasons that are not obvious, inflammatory DCs associate with fibroblasts, potentially stimulating the cells for protease expression. Alternatively, fibroblasts activated by the wound healing process may aberrantly attract and stimulate precursors of inflammatory DCs, inducing an inflammatory process without presenting/producing an autoimmune antigen. The expression of proteases induced in the course of this mechanism may activate precursor effectors, such as the Notch receptors, leading to a number of stimulating effects, including the proliferation of fibroblasts and the production of extracellular matrix components.

There are some limitations of our study. First, our tissue analysis was based on a small patient cohort of only eight keloid-affected patients and five healthy control subjects. We analyzed only lesional and perilesional tissue from keloid-affected patients but not healthy control skin samples from them. Thus, an important first step to support our findings would be to expand the patient cohort to include more samples of keloid tissue, perilesional tissue and healthy control skin from keloid-affected and non-keloid-affected patients. In addition, we received our keloid tissue samples in the context of surgical complete removal. This means that the keloid-affected patients already may receive therapies, such as glucocorticoid therapy, cryotherapy, pressure treatment, laser therapy or radiation therapy. Therefore, it is necessary to compare non-treated keloid tissue to treated keloid tissue, especially in the context of therapy-resistant keloid tissue compared to those showing therapy response under non-surgical treatment.

The strong appearance of activated proteases in keloid lesions implies that these molecules may constitute an ideal target for future therapies. Protease inhibitors have been developed, are in clinical trials and have reached the market [41,42]. It is certainly worthwhile to test such drugs for local injection or systemic therapy during the wound healing process of keloid-affected patients. The spatial analysis of the gradual distribution of cell populations and factors in healthy, perilesional and lesional tissue provides additional insight into new possible pathophysiological processes in the context of keloid development. It must be investigated whether metalloprotease inhibitors show a relevant therapeutic effect. Additionally, if metalloprotease inhibitors show an effect, it must be examined whether they can only help in recurrence prophylaxis, can decrease the progression of keloid tissue or even heal already formed keloid tissue.

Keloid formation is a disfiguring dermatosis, which can reduce the quality of life in affected patients, as the treatment options today are limited. The strong tendency to recurrent appearance and the still unknown mechanisms behind the initiation of this scarring process are major challenges facing dermatologists and today’s medicine. Here, the present data describe an inflammatory environment in keloid scars, which may contribute to the chronic activation of metalloproteases and, therefore, the enormous accumulation of extracellular matrix proteins.

## 4. Materials and Methods

### 4.1. Tissue Samples

We obtained our tissue samples from eight keloid patients (aged 18–47 years) and five unrelated patients undergoing surgical treatments from the Department of Dermatology, University Hospital Erlangen, after approval of the local ethics committee. From each keloid patient, two tissue samples were taken from the active edge of the keloid and from the unaffected perilesional skin around the keloid during an operation. The diagnosis of keloid was based on both the clinical appearance and histopathological examination of the operative specimens. Five samples of healthy skin tissue of keloid-unaffected patients were obtained during non-keloid-related surgeries. This study was approved by the institutional review board of the medical faculty of the University Hospital of Erlangen (approval number 108_8 B) and was conducted following the principles of the Helsinki Declaration in its current version.

### 4.2. Immunohistochemistry

For immunohistochemistry staining, formalin-fixed paraffin-embedded samples were deparaffinized, and antigen retrieval was achieved in tris-EDTA buffer (pH 9) (Agilent Technologies, Santa Clara, CA, USA) for 30 min at 100 °C. For antigen detection, the LSAB method (Dako REAL Detection Systems, (Agilent Technologies, Santa Clara, CA, USA) was used, performed by the Dako Autostainer Plus (Agilent Technologies, Santa Clara, CA, USA).

### 4.3. MAA Sample Preparation

Surgically obtained tissue samples were transported in a 0.9% sodium chloride solution and then deep-frozen at −80 °C in O.C.T. compound (Sakura Finetek, Staufen im Breisgau, Germany) 10.24% polyvinyl alcohol (Carl Roth, Karlsruhe, Germany), 4.26% polyethylene glycol (Carl Roth, Karlsruhe, Germany) and 85.5% non-reactive ingredients). The cryostat tissue was sliced via cryotome Leica SM3050S (Leica Biosystems, Wetzlar, Germany) in a 5 μm wide section and acetone-fixated (Carl Roth, Karlsruhe, Germany) on microscope slides for 10 s. After 10 min of air drying, the tissue slides were stored at −80 °C. For further preparation, the tissue slides were incubated in acetone solution at −20 °C for 10 min and afterwards rehydrated with PBS (phosphate-buffered saline, pH 7.4, Life Technologies, Carlsbad, Germany).

### 4.4. MAA Data Generation

The MAA is a specific technique to locate and illustrate a variety of different antigen structures in a single tissue sample using sequential rounds of immunofluorescence staining. A single tissue slide is placed under a fluorescence microscope, which is part of the MELC robot technology (MelTec, Magdeburg, Germany). This automatic and robotic process encompassed tissue staining via FITC (Fluorescein isothiocyanate)- and PE (Phycoerythrin)-labeled antibodies and a washing process of unbound antibodies with PBS at 20 °C. An inverted wide-field fluorescence microscope (DM IRE2; ×20 air lens; numerical aperture 0.7; (Leica Biosystems, Wetzlar, Germany)) with a cooled CCD camera (KX4; Apogee Imaging Systems, Roseville, CA, USA) acquired the fluorescence images for FITC and PE. Image data recording and coordination of the system components were controlled by software developed by MelTec GmbH & Co KG (Magdeburg, Germany). A soft bleaching process inactivates the fluorochrome of the FITC-/PE-labeled antibodies. Afterwards, the automatic process starts another sequential round of immunofluorescence staining for any given antibody. With the corresponding phase-contrast images, fluorescence images produced after each antibody stain were aligned pixel-wise and were corrected for illumination faults using flat-field correction. The alignment reached a resolution of ±1 pixel. Postbleaching images were subtracted from the following fluorescence tag images. Superimposed images composed an *n* epitope presence in relation to each pixel (approx. 900 × 900 nm^2^ area) of a visual field (2048 × 2048 pixels).

### 4.5. MAA Analysis

The previously acquired fluorescence images were analyzed using the StrataQuest Analysis Software© (TissueGnostics GmbH, Vienna, Austria). Propidium iodide (PI) staining was used for cell nuclei detecting, and a predicted cytoplasm seam as a cellular mask was added by a prefabricated algorithm. By superimposing PI-stained tissue images with the individual FITC-/PE-labeled antibody tissue images, both the expression of a specific antigen and the exact cell number expressing a specific antigen were analyzed. Afterwards, we adapt manually for each slide and antibody the thresholds for cell nuclei size detection, cellular mask detection, positive antigen expression, and we remove artefacts that may affect the results. Thus, we can determine the total cell density per square millimeter, the cell density per square millimeter expressing a specific antigen and the relative sum intensity of a specific antigen expression in a single tissue section. Furthermore, by superimposing multiple tissue images, we can illustrate and analyze the spatial arrangement of different antigen expression patterns and cell subpopulations to each other.

The spatial distribution of cell populations was characterized both qualitatively and quantitatively using Ripley’s K functions and their normalized versions, Bessag’s L functions. Those approaches make it possible to describe the spatial distribution of one or more cell populations at multiple scales, i.e., in circular neighborhoods of various radii. We could assess (a) whether the cells from one population were randomly distributed, clustered or dispersed and (b) whether they were spatially segregated. By summing the values of the K or L functions, we could also obtain quantitative measurements, based on which we could run statistical tests to distinguish between cases.

### 4.6. MAA and IHC Antibodies

The best working dilutions of the antibodies for the MAA analysis were determined in initial calibration runs, adjusted if necessary, and tested again (Table 1).

### 4.7. Statistical Analysis and Heat Maps

GraphPad Prism (Version 8, GraphPad Software, San Diego, CA, USA) was used for heat maps and statistical analysis (one- and two-way ANOVA and Tuckey’s, Bonferroni’s and Dunnett’s test for multiple comparison tests). Data were expressed as mean ± standard error of the mean unless otherwise stated.

## Figures and Tables

**Table 1:** Antibodies used for MAA and IHC.

Antibody	Clone	Company	Dilution
ADAM10-PE	SHM14	BioLegend	1:40
ADAM10	1E12	Helmholtz Zetrum München	1:100
ADAM17-FITC	111633	R&D Systems	1:40
ADAM17	1B7	Helmholtz Zetrum München	1:100
CD1a-PE	NA1/34	Dako	1:40
CD1a	EP3622	Cell Marque	1:200
CD3-PE	UCHT1	ImmunoTools	1:40
CD4-PE	OKT-4	ImmunoTools	1:40
CD5-PE	LT1	ImmunoTools	1:20
CD6-PE	HI210	ImmunoTools	1:20
CD7-PE	LT7	ImmunoTools	1:20
CD8-PE	HIT8a	ImmunoTools	1:40
CD9-PE	HI9a	ImmunoTools	1:40
CD10-FITC	LT10	ImmunoTools	1:10
CD10	56C6	DCS	1:250
CD11a-PE	HI111	ImmunoTools	1:40
CD11c-FITC	MJ4-27G12.4.6	Miltenyi Biotec	1:10
CD11c	5D11	DCS	1:100
CD14-PE	18D11	ImmunoTools	1:40
CD14	EPR3653	DCS	1:100
CD20-FITC	LT20	ImmunoTools	1:20
CD29-FITC	HI29a	ImmunoTools	1:40
CD38-PE	HIT2	ImmunoTools	1:20
CD39-PE	MZ18-23C8	Miltenyi Biotec	1:10
CD40-PE	HI40a	ImmunoTools	1:40
CD44-PE	IM7	ImmunoTools	1:40
CD45-PE	HI30	ImmunoTools	1:40
CD45RA-PE	HI100	ImmunoTools	1:20
CD45RO-PE	UCHL1	ImmunoTools	1:20
CD54-PE	1H4	ImmunoTools	1:40
CD56-PE	B-A19	ImmunoTools	1:40
CD58-PE	HI58a	ImmunoTools	120
CD63-FITC	MEM-259	BioLegend	1:40
CD68-FITC	KP1	Dako	1:40
CD69-FITC	IT8G1	ImmunoTools	1:20
CD90-FITC	DG3	Miltenyi Biotec	1:40
CD95-FITC	LT95	ImmunoTools	1:20
CD107a-FITC	H4A3	BD Pharmingen	1:40
CD117-FITC	7	Novus Biologicals	1:20
CD169-FITC	7-239	AbD Serotec	1:40
CD276-FITC	FM276	Miltenyi Biotec	1:20
Collagen IV-FITC	5K134	Biomol	1:200
Fibroblast-FITC	REA165	Miltenyi Biotec	1:10
HLA-ABC-PE	W6/32	ImmunoTools	1:40
HLA-DR-PE	LT-DR	ImmunoTools	1:40
Notch1-FITC	mN1A	abcam	1:20
Notch3-PE	MHN3-21	BioLegend	1:10
Propidium iodid		Genaxxon Bioscience	1:1000
Vimentin-FITC	V9	Santa Cruz Biotechnology	1:100

## Data Availability

Data are contained within the article or supplementary material.

## Supplementary Materials

The following are available online at https://www.mdpi.com/article/10.3390/ijms22179417/s1.
