# Supplementary material for: Multi-Antigen Imaging Reveals Inflammatory DC, ADAM17 and Neprilysin as Effectors in Keloid Formation
Source: Int J Mol Sci. 2021 Aug 30;22(17):9417. doi: 10.3390/ijms22179417 (PMC8430546; doi:10.3390/ijms22179417)
Supplement: Supplementary file 1 [file ijms-22-09417-s001.zip › ijms-1341306-supplementary.pdf]

SUPPLEMENTARY MATERIAL

Supplementary Figures

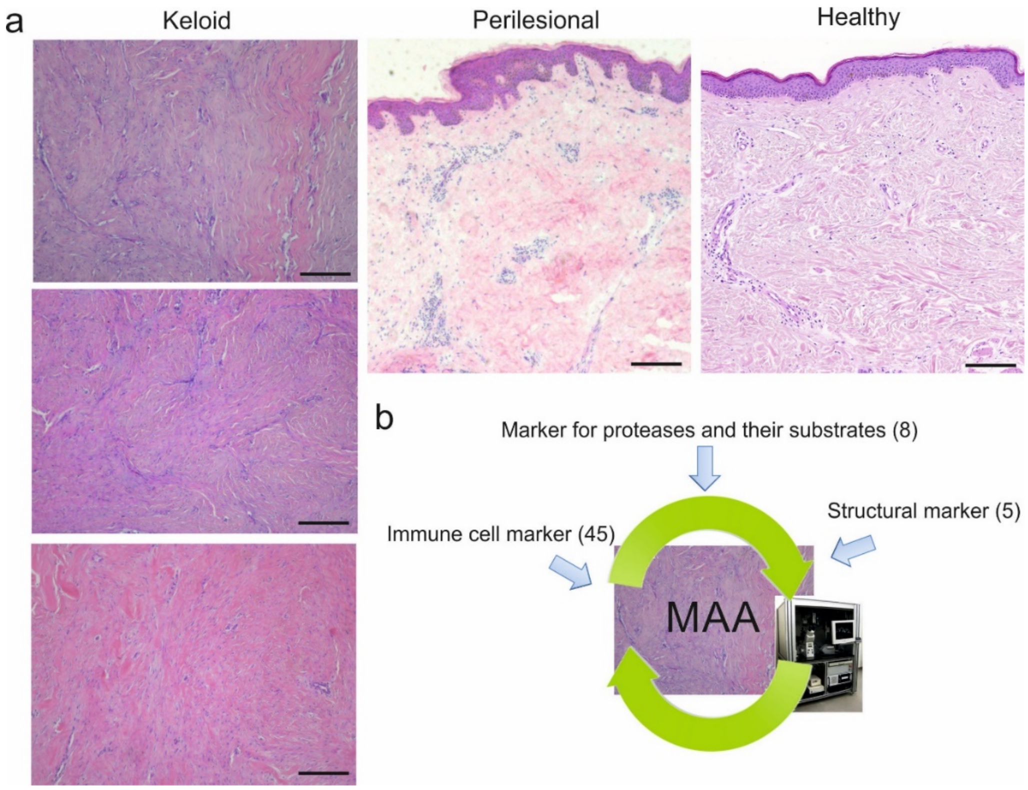

Figure S1. H&E staining of analyzed tissue by MAA.

(a) Representative H&E stainings of keloid, perilesional and control tissue. Scale bars = 200 µm. (b) Simplified concept of the MAA-technology. MAA visualizes up to 100 and more individual antigens on one single tissue section or portray individual cells, while preserving the sub-cellular structure and organization of the sample. The MAA is a fully automated cyclic process of (i) staining by fluorescent antibodies, (ii) imaging and (iii) photobleaching of the fluorochrome. For the analyses of keloid tissue a total number of 58 different antibodies were used.

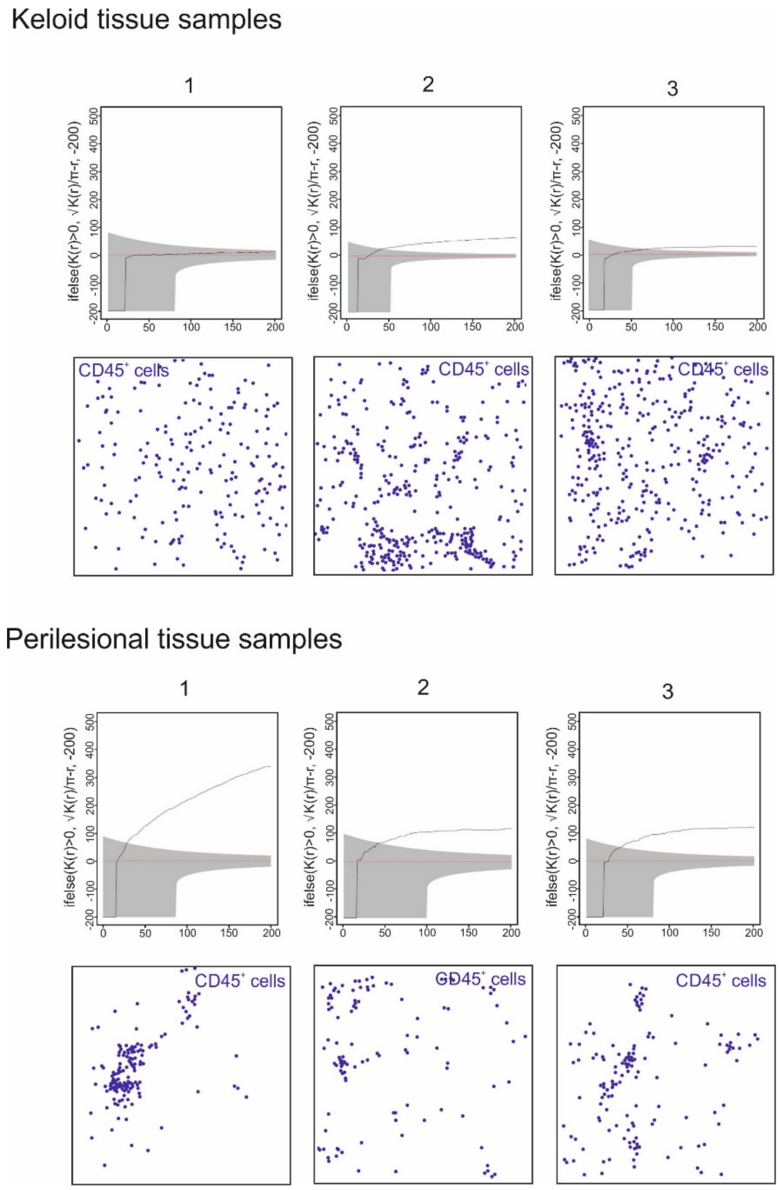

Figure S2. Diffuse spatial distribution of CD45⁺ cells in keloid tissue.

The spatial distribution of the CD45+ cells (blue dots in the second rows) were assessed with Bessag's L functions (solid black line in the first row plots). For each sample the acceptance envelope (grey) gives the range of L values for which the cell distribution is not significantly different from complete randomness. The further the L function is from the grey envelope, the further the cell distribution is from spatial randomness (dotted red line). r (on X axis) is in pixels.

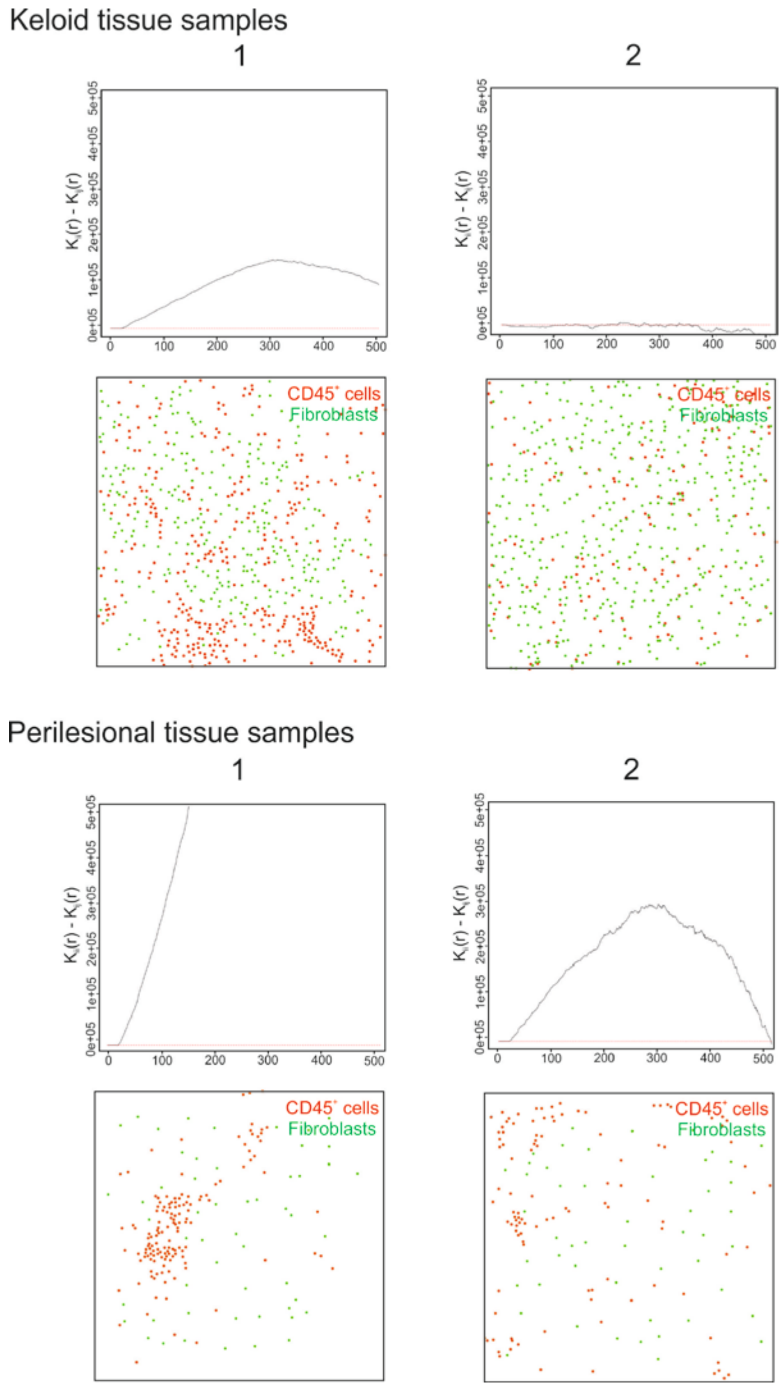

Figure S3. High association of immune cells and fibroblasts in keloid.

Analysis of the spatial segregation of the immune cell (red) and fibroblast (green) populations in lesional and perilesional tissue. K_{ii} - K_{ij}, for i = CD45 and j = fibroblast, was used to assess the spatial segregation of the two populations. r (on X axis) is in pixels. $CD45^+$ cells are much more segregated in perilesional tissue than in lesional tissue.

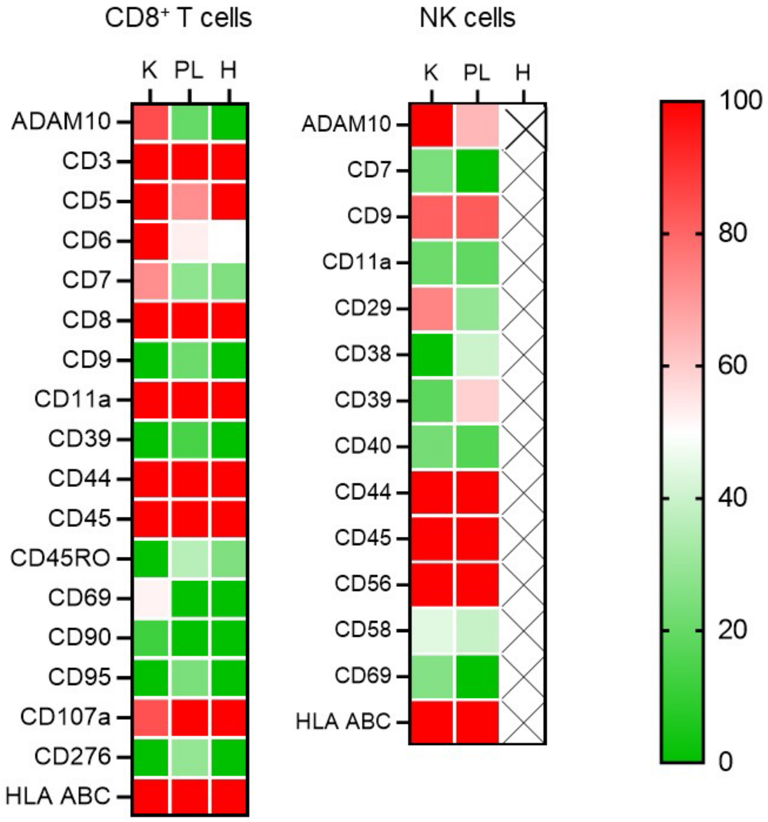

Figure S4. Expression profiling of immune subtypes.

Heat maps of $CD8^+$ T cells and NK cells display the expression intensity of indicated markers in keloid (K), perilesional (PL) and control (H) skin sections. The color code from red (100) to green (0) shows the expression intensity of the antigens in % in all cells of a certain immune subtype.

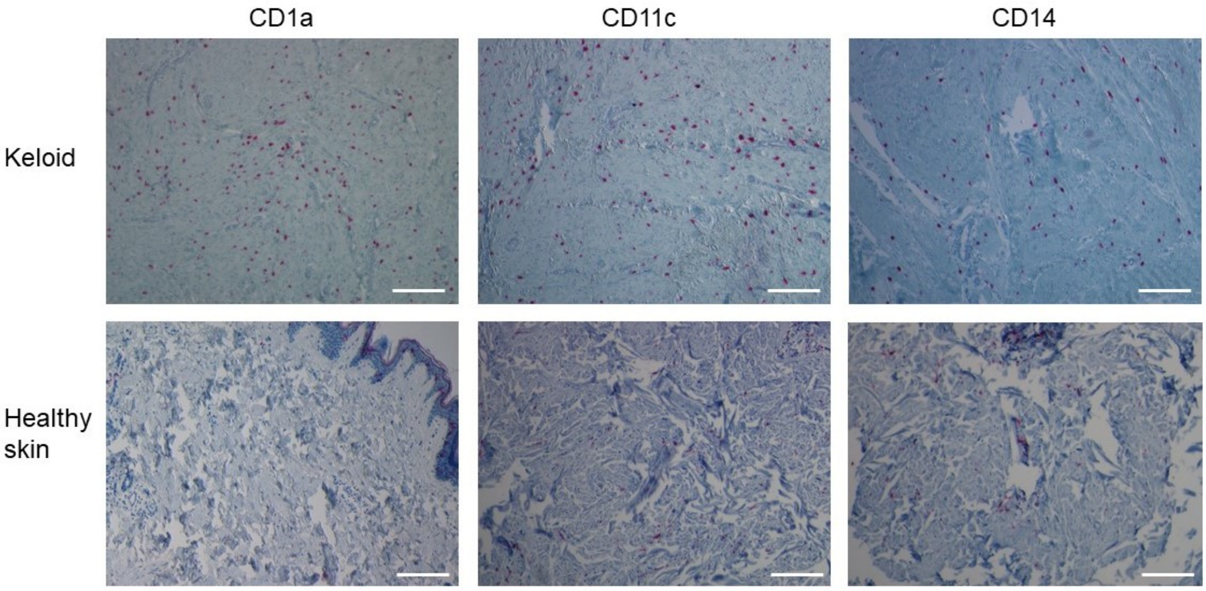

Figure S5. Staining of CD1a, CD11c and CD14 in keloid and healthy skin.

Immunohistochemical staining of cell surface marker CD1a, CD11c and CD14 in lesional and control tissue. Scale bars = 100 µm.

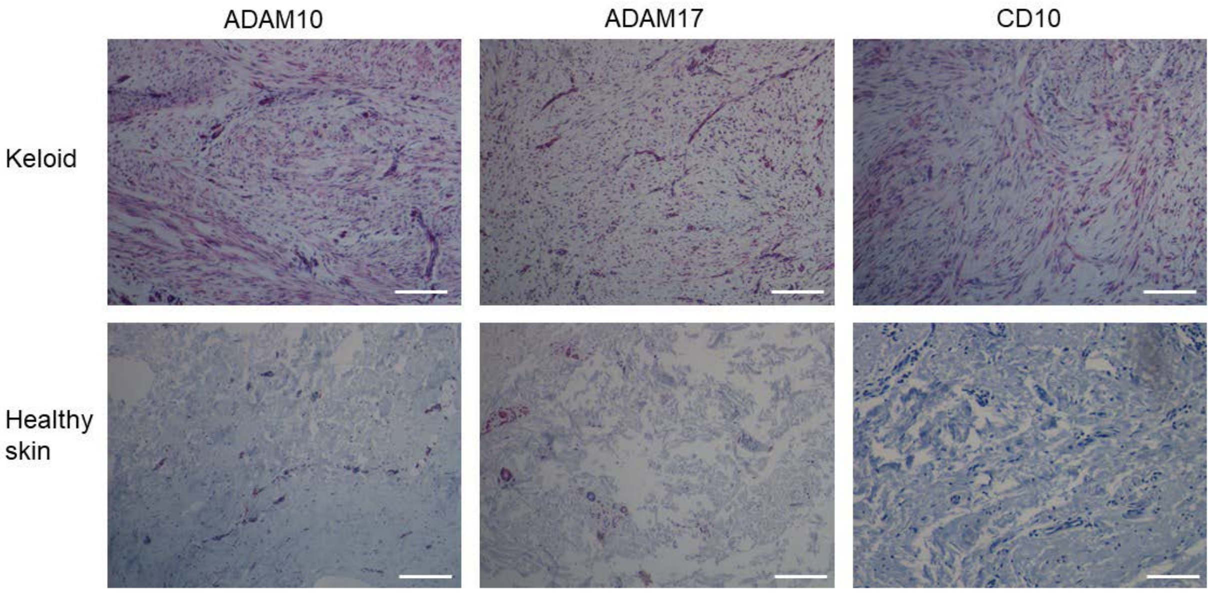

Figure S6. Differential expression of proteases in lesional and healthy tissue.

Representative images showing the ADAM10, ADAM17 und CD10 expression in keloid and control tissue sections by conventional immunohistochemistry. Scale bars = 100 µm.

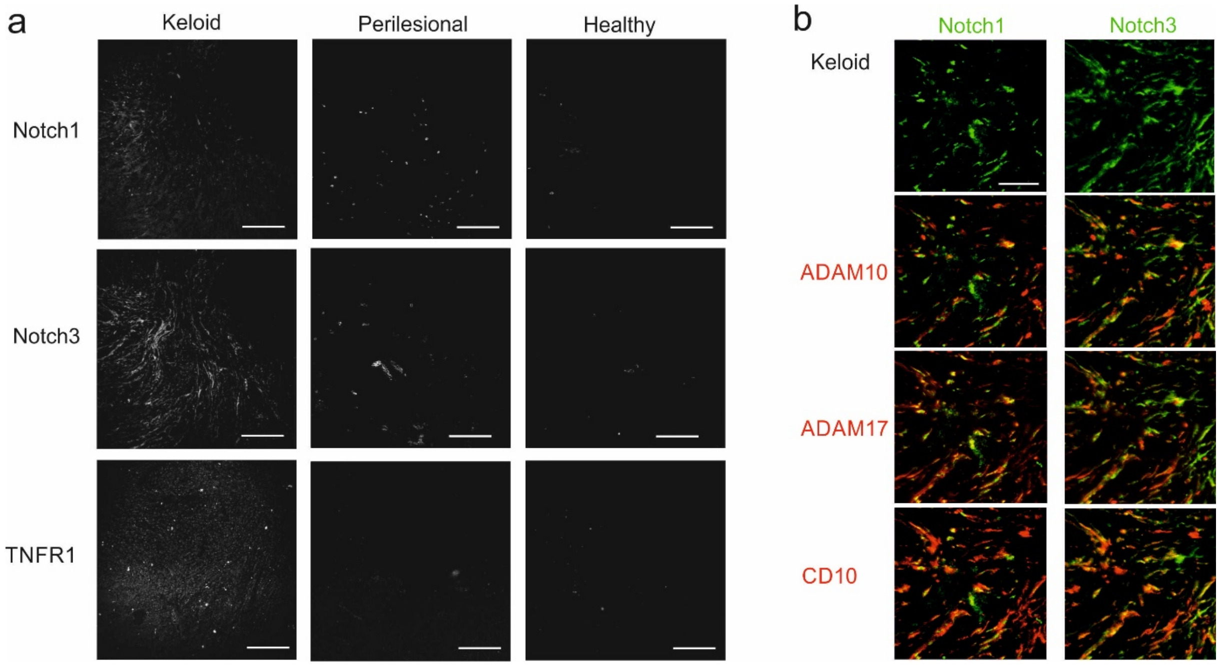

Figure S7. Increased levels of substrates of metalloproteases and their co-localization in keloid tissue.

(a) Notch1, Notch3 and TNFR1 expression are shown for keloid, perilesional and healthy tissue. Scale bars = 200 µm. (b) Overlay images and images of labeled markers indicate the co-localization of the Notch proteins with its proteases (ADAM10, ADAM17 and CD10). Scale bars = 50 µm.
